# High prevalence of ultra‐short celiac disease in children with low anti‐tissue transglutaminase immunoglobulin A levels: The risk of misdiagnosis without bulb biopsy

**DOI:** 10.1002/jpn3.70464

**Published:** 2026-05-25

**Authors:** Antonio Pizzol, Eleonora Saraceno, Anna Opramolla, Laura Giugliano, Cristina Chiadò, Tilde Manetta, Gabriella Canavese, Enrico Falco, Michele Pinon, Pier Luigi Calvo

**Affiliations:** ^1^ Pediatric Gastroenterology Unit, Regina Margherita Children's Hospital, Azienda Ospedaliera‐Universitaria Città della Salute e della Scienza di Torino Turin Italy; ^2^ Department of Laboratory Medicine Città della Salute e della Scienza University Hospital Turin Italy; ^3^ Pathology Unit, Department of Medical Sciences University of Turin Turin Italy

**Keywords:** duodenal bulb sampling, guidelines adhesion, pediatric celiac disease, potential celiac disease

## Abstract

**Objectives:**

Despite recent advances in diagnostics, celiac disease (CD) diagnosis remains challenging. Ultra‐short celiac disease (USCD) has been proposed as a distinct phenotype characterized by villous atrophy confined to the duodenal bulb, positive celiac serology, and clinical features consistent with CD. This study aimed to assess the impact of adherence to European Society for Pediatric Gastroenterology, Hepatology and Nutrition (ESPGHAN) guidelines, including duodenal bulb biopsy, in the diagnostic process of CD, and to investigate whether omission of bulb sampling may have led to misclassification of potential celiac disease (PCD).

**Methods:**

We conducted a retrospective, observational study of pediatric patients evaluated for CD between June 2017 and December 2024 at a tertiary‐care center. Patients undergoing biopsy‐based diagnosis were stratified by biopsy protocol: distal duodenum (DII) versus distal duodenum plus bulb (DII + Bulb). A logistic regression model using tissue transglutaminase immunoglobulin A (TTG‐IgA) levels as predictor estimated the likelihood of missed USCD among PCD cases in the DII group.

**Results:**

Out of 474 patients undergoing endoscopy, 361 had TTG‐IgA <10 × ULN. Of these, 24 were in DII group and 337 in DII + Bulb group. In the latter, 140 (41.5%) were classified as classic CD, 54 (16%) as PCD, and 143 (42.4%) as USCD. PCD detection dramatically dropped after full adherence to guidelines. The logistic model identified seven of nine PCD patients in DII group as likely USCD.

**Conclusions:**

Systematic duodenal bulb biopsy significantly improves USCD detection in patients with low TTG‐IgA levels. PCD cases diagnosed without bulb sampling warrant reassessment, especially during transition to adult care.

## Abstract (highlights)

**What is Known:**

- A no‐biopsy approach for the diagnosis of celiac disease (CD) is increasingly adopted in pediatric practice.
- Biopsy remains essential for diagnosis in patients with tissue transglutaminase immunoglobulin A (TTG‐IgA) levels below 10 times upper limit of normal (ULN).
- An emerging body of literature recognizes ultra‐short celiac disease (USCD) as a form of CD with histological damage limited to the duodenal bulb.

**What is New:**

- In patients with TTG‐IgA <10 × ULN, the detection of USCD is steadily increasing when duodenal bulb biopsies are included, with the highest yield observed in those with lower TTG‐IgA levels.
- Duodenal bulb biopsies are not consistently performed during endoscopy, even in high‐volume specialized centers, increasing the risk of false‐negative diagnoses.
- At the time of transition to adult care, patients with potential CD—especially those diagnosed without duodenal bulb biopsies—should be re‐evaluated endoscopically.

## Graphical abstract

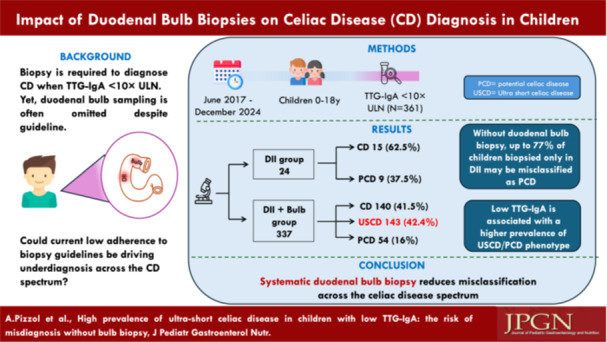

## 1 INTRODUCTION

Celiac disease (CD) is a systemic immune‐mediated enteropathy triggered by dietary gluten in genetically predisposed individuals.

It affects approximately 1% of the global population, with a reported prevalence of 1.65% among the pediatric population in Italy.^1^

Diagnosis relies on a combination of serological and histological criteria, as defined by current guidelines issued by European Society for Pediatric Gastroenterology, Hepatology and Nutrition (ESPGHAN, 2020)^2^ and the American College of Gastroenterology (ACG, 2023).^3^ Since their introduction, diagnostic strategies have markedly evolved, allowing a non‐biopsy approach in children with serum anti–tissue transglutaminase immunoglobulin A (TTG‐IgA) ≥10× the upper limit of normal (ULN) and positive endomysial antibodies (EMA) on a separate blood sample. However, histological confirmation remains essential for patients with TTG‐IgA <10× ULN.^2^

Traditionally, CD has been considered to predominantly affect distal duodenum (DII). However, duodenal bulb has been increasingly recognized as a critical site of early mucosal damage,^4^, ^5^ and in up to 14% of CD cases, it may represent the exclusive site of villous atrophy.^6^, ^7^, ^8^, ^9^, ^10^, ^11^

Ultra‐short celiac disease (USCD) has been proposed as a distinct phenotype, first systematically described in 2016, characterized by villous atrophy confined to the duodenal bulb, in the presence of positive celiac serology and clinical features consistent with CD.^12^, ^13^ Clinically, patients with USCD have been associated with younger age at diagnosis and lower TTG‐IgA levels, potentially reflecting earlier detection within the disease course.^13^

Accordingly, both the 2012 ESPGHAN^14^ and 2013 AGA guidelines^15^—as well as their respective updates in 2020^2^ and 2023^3^—clearly endorse the recommendation to obtain at least four biopsies from the distal duodenum and at least one from the duodenal bulb in the evaluation of suspected CD across all age groups.

While substantial advancements have been made in serologic and histologic diagnostics, CD remains a highly prevalent yet underdiagnosed condition. This is attributable not only to the high prevalence of nonclassical or asymptomatic presentations, but also to the persistently suboptimal adherence to recommended biopsy protocol.^6^, ^16^, ^17^

Within this framework, the under recognition of USCD may represent a key contributor to the diagnostic gap in CD.

This study aims to assess the impact of duodenal bulb biopsy on diagnostic outcomes and to characterize the clinical and serological features of USCD in comparison to CD. Furthermore, we investigate whether omission of duodenal bulb sampling in the recent past may have resulted in the misclassification of potential celiac disease (PCD) in patients who could have fulfilled criteria for USCD if extended biopsy had been performed at the time of the diagnosis. Ultimately, this study evaluates whether the expanded biopsy protocol including biopsies from both the distal and bulb duodenum may reduce the risk of misdiagnosis within the CD spectrum.

## 2 METHODS

### 2.1 Ethics statement

Institutional Review Board approval was obtained from the Azienda Ospedaliero‐Universitaria Città della Salute e della Scienza di Torino (project N. 00342/2020). The requirement for informed consent was waived by the Institutional Review Board due to the retrospective nature of the study.

### 2.2 Study design and participants

We conducted a retrospective, observational cross‐sectional study on patients under 18 years of age who underwent esophagogastroduodenoscopy (EGD) for suspected CD between June 2017 and December 2024 at a single tertiary‐care pediatric center in northern Italy.

Clinical, laboratory, and histological data were extracted from electronic medical records (EMRs) by trained investigators to ensure consistency and accuracy. All data were anonymized prior to analysis.

### 2.3 Histological methods

Biopsy specimens were assessed by a dedicated team of experienced pathologists, each with a minimum of 5 years of diagnostic practice. The main histological features assessed included villous atrophy, crypt hyperplasia, increased intraepithelial lymphocytes (IELs), and lamina propria inflammation. Multiple 2.5 μm sections were prepared for each biopsy and stained with hematoxylin and eosin (H&E) to accurately evaluate the degree of villous atrophy and the villous‐to‐crypt (V:C) ratio. Ratios <2 in DII region and <1 in duodenal bulb were considered abnormal.^18^ To allow objective quantification of intraepithelial T lymphocytes, all biopsies were immunostained with the CD3 antibody. IEL counts exceeding 30 lymphocytes per 100 epithelial cells were considered elevated. CD was diagnosed in cases showing Marsh‐Oberhuber grade 3 lesions, corresponding to Corazza‐Villanacci grade B1 or B2.^19^

### 2.4 Inclusion and exclusion criteria

Eligibility criteria included the availability of serological data and histological evaluation performed on either DII alone or both DII and duodenal bulb.

Exclusion criteria were: prior confirmed diagnosis of CD, pre‐existing gluten‐free diet at the time of evaluation, inadequate biopsy samples preventing proper histological assessment and clinical or endoscopic evidence of an alternative non‐celiac enteropathy accounting for the presentation (e.g., *Helicobacter pylori* infection or inflammatory bowel disease).

Patients were also excluded if serology was performed using chemiluminescence assays, if celiac serology was negative with normal or non‐diagnostic histological findings on duodenal biopsy (e.g., Marsh 1), if TTG‐IgA results were missing, or in case of selective IgA deficiency.

### 2.5 Data abstraction

Age, sex, and clinical presentation were recorded at the time of evaluation.

TTG‐IgA levels, obtained from laboratory records, were measured using ELISA‐based assays and expressed as multiples of the ULN.

For patients diagnosed prior to 2020, CD diagnosis was based on 2012 ESPGHAN guidelines^14^ and all asymptomatic patients with positivity in TTG‐IgA levels underwent endoscopic evaluation. An extended duodenal bulb biopsy protocol was introduced in May 2018 and progressively adopted. Starting in 2020, the updated 2020 ESPGHAN guidelines^2^ were implemented, resulting in the adoption of both the non‐biopsy approach for selected patients and the routine use of the extended duodenal bulb biopsy protocol in our center.

USCD was defined by the presence of both histological alterations of CD confined to duodenal bulb (Marsh ≥ 2) and normal or Marsh 1 in distal duodenum, in the presence of positive celiac serology.

PCD was identified in patients with positive celiac serology but normal or Marsh 1 in both distal and bulb duodenum.

According to the biopsy protocol used, patients were classified into two biopsy groups: (1) isolated distal duodenum sampling (DII group) or (2) combined distal duodenum and duodenal bulb sampling (DII + Bulb group).

### 2.6 Statistical analysis

Clinical characterization of CD and USCD was performed by analyzing the distribution of presenting symptoms in all patients within the DII + Bulb group. Comparative analyses of age at diagnosis and TTG‐IgA levels between CD and USCD within DII + Bulb group were restricted to patients with TTG‐IgA <10× ULN to minimize bias from the progressive introduction of the non‐biopsy approach.

Similarly, comparative analyses between the DII and DII + Bulb groups (focused on patients with USCD or PCD and TTG‐IgA <10× ULN) were designed to ensure reliability across biopsy protocols and diagnostic eras.

Categorical variables were expressed as frequency (percentages). The normality of continuous variables was assessed using the Shapiro–Wilk test. Continuous variables were summarized using mean (standard deviation [SD]) for normally distributed data and median (interquartile range [IQR]) for non‐normally distributed data.

For comparisons, independent *t*‐tests were used for normally distributed variables, while Wilcoxon Rank Sum Test were applied for non‐normally distributed variables. Categorical data were compared using the Chi‐square test. A two‐sided *p*‐value < 0.05 was considered statistically significant.

A detailed description of the statistical models, including the logistic regression analyses used to evaluate TTG‐IgA as a predictor of CD versus USCD and to estimate the likelihood of missed USCD diagnoses in the DII group, is provided in the Supplemental File 1.

All statistical analysis was performed in R version 4.4.0 *(Boston, MA*) (2024‐04‐24) using R Statistical Software.

## 3 RESULTS

A total of 474 pediatric patients underwent endoscopic evaluation due to clinical suspicion of CD. After applying the exclusion criteria, 419 patients were included in the final analysis; specifically, 57 were assigned to the DII group and 362 to the DII + Bulb group (Table 1).

**Table 1:** Demographic and clinical characteristics of the study population, stratified by diagnosis (CD, USCD, and PCD).

Diagnosis	*N*	Age at diagnosis (median, IQR)	Male (*N*, %)	TTG‐IgA levels (median, IQR)
Overall	419	9 (6–12)	170 (40.6%)	3.29 (2–6.2)
CD	211	10 (6–12)	95 (45%)	4.71 (2.46–10.5)
USCD	143	9 (6–12)	40 (28%)	2.57 (1.71–4.29)
PCD	65	7 (4–10)	35 (53.8%)	2.54 (1.7–4)

*Note*: Continuous variables are reported as median (IQR); categorical variables are reported as number (percentage).

Abbreviations: CD, celiac disease; IQR, interquartile range; PCD, potential celiac disease; TTG‐IgA, tissue transglutaminase immunoglobulin G; USCD, ultra‐short celiac disease.

The median age of included patients was 9 years (IQR: 6–12), with a female‐to‐male ratio of 2:1. Table S1 summarizes the primary presenting symptoms of patients diagnosed with CD and USCD in DII + Bulb group, among those with available clinical history.

For the purpose of evaluating the diagnostic sensitivity of intestinal biopsy sites according to current ESPGHAN guidelines,^2^ all subsequent analysis were restricted to patients with TTG‐IgA levels <10× ULN, regardless of year of diagnosis.

Within the TTG‐IgA <10× ULN subgroup, 24 patients were included in the DII group and 337 in the DII + Bulb group. In the DII + Bulb group, CD was diagnosed in 41.5% (*n* = 140), USCD in 42.4% (*n* = 143), and PCD in 16% (*n* = 54). Among patients in the DII group, CD was diagnosed in 62.5% (*n* = 15), while the remaining 37.5% (*n* = 9) were classified as PCD.

A significant difference in sex distribution was observed among diagnostic groups (*p* < 0.001). Male patients were more prevalent in the PCD (53%) and CD (47.7%) groups, whereas females predominated in the USCD group (72%).

Notably, the proportion of EGD including duodenal bulb biopsies increased progressively over the study period, from 0/17 (0%) in 2017, to 15/70 (21%) in 2018, 76/80 (95%) in 2019, and reached full adherence (100%) from 2020 onwards.

### 3.1 DII + Bulb group analysis in patients with TTG‐IgA levels <10× ULN

**Figure 1:**
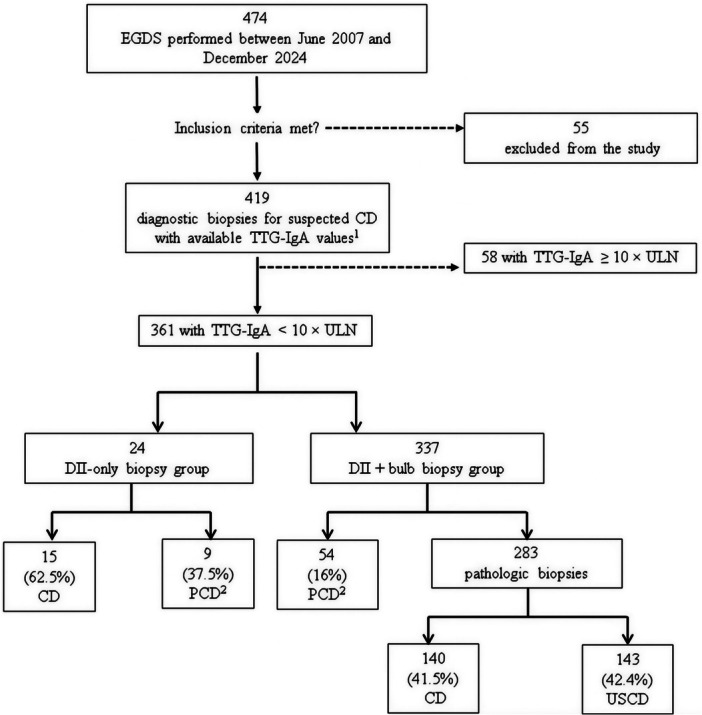
Flowchart of patient selection and biopsy outcomes. Of 474 patients undergoing endoscopy for suspected CD, 419 with available TTG‐IgA values met inclusion criteria. Patients with TTG‐IgA ≥10× ULN were excluded from subsequent analysis. The remaining subgroup with TTG‐IgA <10× ULN was stratified by biopsy protocol (DII vs. DII + Bulb) and classified as CD, USCD, or PCD. Percentages refer to the distribution within each biopsy group (DII or DII + Bulb), not to the overall cohort. ^1^Including patients from the period prior to full adherence to the 2020 ESPGHAN guidelines.^2^ ^2^PCD defined by normal or Marsh 1 histology in both distal and bulb duodenum. CD, celiac disease; DII, distal duodenum; EGDs, esophagogastroduodenoscopies; PCD, potential celiac disease; TTG‐IgA, tissue transglutaminase immunoglobulin G; ULN, upper limit of normal; USCD, ultra‐short celiac disease*.*

Within this subgroup (Figure 1), no statistically significant difference in age at diagnosis was observed between CD and USCD patients (*p* = 0.6). A significant association between sex and diagnosis was observed (*p* = 0.002), with females representing 72% (103/143) of the USCD group and 54% (75/140) of the CD group.

Median TTG‐IgA levels were significantly lower in USCD patients (2.57 [IQR 2.29–6.43] × ULN) compared to CD patients (3.775 [IQR 2.29–6.43] × ULN; *p* < 0.001) (Figure S1).

### 3.2 Impact of biopsy sampling strategy on diagnostic distribution

Within patients with TTG‐IgA <10× ULN, the distribution of histological diagnoses differed significantly according to biopsy sampling strategy (*p* < 0.001) (Figure 1).

Specifically, in the DII + Bulb group, USCD accounted for 42.4% of cases, a frequency comparable to that of classic CD (41.5%). In contrast, PCD diagnoses dropped from 37.5% in the DII group to 16% in the DII + Bulb group.

Importantly, TTG‐IgA levels did not significantly differ between USCD and PCD cases (*p* = 0.32).

### 3.3 Association between TTG‐IgA range and diagnostic outcome

**Figure 2:**
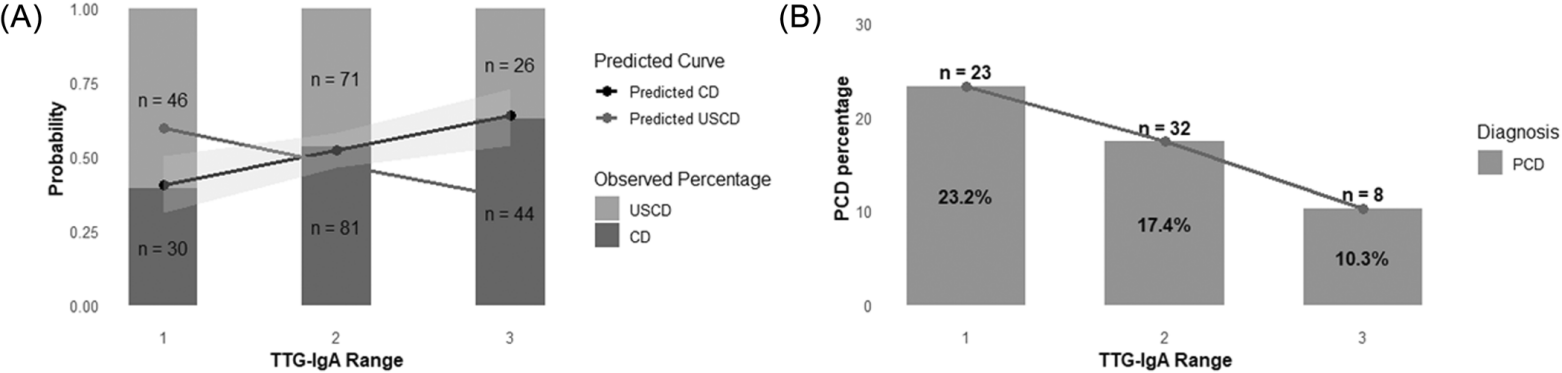
(A) Observed and predicted probability of CD and USCD across TTG‐IgA ranges in patients with TTG‐IgA <10× ULN. Stacked bars show observed proportions; lines represent predicted probabilities from logistic regression. The shaded area represents the 95% CI. Increasing TTG‐IgA range was significantly associated with CD (OR: 1.69; 95% CI: 1.23–2.33; *p* = 0.0014). (B) Proportion of PCD diagnoses by TTG‐IgA range. A decreasing trend was noted: 23.2% (*n* = 23) in range 1, 17.4% (*n* = 32) in range 2, and 10.3% (*n* = 8) in range 3. TTG‐IgA ranges were defined as follows: range 1 = <2× ULN; range 2 = 2–5× ULN; range 3 = 5–10× ULN. CD, celiac disease; CI, confidence interval; OR, odds ratio; PCD, potential celiac disease; TTG‐IgA, tissue transglutaminase immunoglobulin A; ULN, upper limit of normal; USCD, ultra‐short celiac disease.

Analysis of diagnostic distribution across increasing TTG‐IgA ranges showed a progressive increase in the proportion of CD diagnoses relative to USCD (Figure 2A). Conversely, the proportion of PCD diagnosis decreased with higher TTG‐IgA levels (Figure 2B).

A binary logistic regression model confirmed a statistically significant association between higher TTG‐IgA range and the likelihood of receiving a CD diagnosis.

The odds ratio (OR) was 1.61 (95% CI: 1.16–2.27; *p* = 0.005), indicating that for each incremental increase in TTG‐IgA range, the odds of CD diagnosis increased by 61%.

### 3.4 Predictive reclassification and follow‐up outcomes of PCD patients in DII group

To explore potential misclassification related to limited biopsy sampling, a predictive logistic regression model based on TTG‐IgA levels was applied to patients classified as PCD in DII group.

Based on the model's predicted probabilities, seven of the nine patients initially defined as PCD were reclassified as USCD.

Five‐year follow‐up data further supported this hypothesis: two patients underwent a second endoscopy including duodenal bulb biopsies (DII + Bulb), confirming a CD diagnosis. One patient developed TTG‐IgA positivity ≥10× ULN, meeting criteria for a no‐biopsy diagnosis of CD. Four patients were initiated on a gluten‐free diet due to persistent gastrointestinal symptoms, achieving clinical and serologic improvement. One patient spontaneously normalized TTG‐IgA without dietary intervention. One patient was lost to follow‐up, with no recent data available.

## 4 DISCUSSION

Our retrospective study aimed to evaluate the impact of biopsy sampling strategies on the diagnosis of CD and USCD in a pediatric cohort from June 2017 to December 2024.

During this period, the initial biopsy protocol targeting only DII was progressively expanded following the implementation of the 2020 ESPGHAN guidelines.^2^ This led to the routine inclusion of duodenal bulb biopsies (DII + Bulb) and the introduction of a non‐biopsy diagnostic approach for patients with TTG‐IgA ≥10× ULN and positive EMA on a separate blood sample. Compliance with the updated guidelines increased over the study period, reaching full adherence by 2020, as reflected in the temporal distribution of bulb sampling in our cohort.

To minimize potential bias, we focused our analysis on patients with TTG‐IgA <10× ULN, for whom histology remained mandatory throughout the study period.

Specifically, within DII + Bulb group the incidence of USCD was 39.5%, higher than previously reported.^7^, ^13^, ^20^, ^21^, ^22^ No difference in age at diagnosis emerged between CD and USCD, although USCD showed a female predominance and lower median TTG‐IgA levels, consistent with previous reports.^13^, ^23^

Notably, PCD cases accounted for 16% of this subgroup, in line with existing literature and supporting the overall reliability of our findings.^24^

In the context of an increasingly serology‐driven diagnostic approach to CD, our analysis highlights a strong association between antibody level and diagnostic classification. A progressive serological gradient was evident across diagnostic categories: the proportion of CD diagnoses increased progressively with higher TTG‐IgA levels, whereas both USCD and PCD were more frequently at lower rates (Figure 2A,B). Our binary logistic regression model further demonstrated that each incremental rise in TTG‐IgA range increased the likelihood of CD diagnosis by 61% (Figure 2A), thus reinforcing the diagnostic role of extended duodenal sampling particularly at lower antibody levels.

Consistently, among patients with TTG‐IgA <10× ULN no significant difference in serological levels was observed between USCD and PCD.

Comparison between biopsy protocols in patients with TTG‐IgA <10× ULN further emphasized the importance of duodenal bulb sampling. In DII group, the absence of USCD cases coincided with a higher proportion of PCD diagnoses compared to the DII + Bulb group (37.5% vs. 15.7%), exceeding published estimates.^24^

Given the critical role of bulb biopsies in detecting USCD and the overlapping serological profiles between USCD and PCD, it is plausible that the omission of duodenal bulb sampling may have contributed to under‐recognition of isolated bulb pathology, leading to initial misclassification of some USCD cases as PCD.

To explore this hypothesis, we applied a logistic regression model validated in the DII + Bulb cohort to patients initially classified as PCD within DII group. The model re‐classified seven out of nine as USCD. Longitudinal follow‐up over 5 years supported these predictions: three patients developed biopsy‐proven CD, while in four others, CD could reasonably be presumed based on sustained clinical improvement on a gluten‐free diet, although not formally confirmed.

These findings underscore how subtle histological changes may go unrecognized without adequate duodenal bulb sampling, potentially leading to misclassification—especially in the early stages of the disease—and suboptimal management.

As reported by Raju et al.,^6^ incorporating D1 sampling into the diagnostic work‐up for CD increases diagnostic yield by 9.7%. Nevertheless, despite endorsement from the American College of Gastroenterology^3^ and the British Society of Gastroenterology,^25^ adherence to recommended biopsy protocols remains suboptimal, particularly outside of tertiary referral centers, with compliance rates ranging from 37% to 39.5%.^17^, ^26^

The reluctance in adopting bulb sampling in routine clinical practices may stem from the increased histopathological complexity, owing to the presence of Brunner glands, gastric metaplasia, and duodenitis, which may obscure villous architecture and complicate the V:C ratio.^13^ Therefore, careful orientation of bulb specimens and comparison with distal duodenal samples are essential steps to avoid overinterpretation. Based on these considerations, we adopted a conservative approach and defined a V:C ratio <1 as indicative of mucosal damage in this region. For DII samples, instead, we followed the 2020 ESPGHAN recommendations,^2^ considering a V:C ratio <2 as pathological. These methodological refinements are particularly relevant for cases with mild or patchy lesions, where duodenal bulb involvement may critically influence diagnostic reclassification.

Additional practical barriers, including increased processing costs and longer turnaround times, may further limit routine bulb sampling.^27^

In pediatric centers, procedural heterogeneity—often due to mixed adult–pediatric or surgeon endoscopy teams—further contributes to variability in sampling practices. Across Europe, up to 45% of centers rely on such mixed models^28^, ^29^ and fewer than four duodenal biopsies are obtained in up to 25% of CD cases.^30^

This context raises a crucial diagnostic question: given the increasing recognition of USCD when extended biopsy protocols are correctly implemented, how reliable is the classification of PCD in the absence of systematic bulb biopsies?

According to the 2020 ESPGHAN guidelines,^2^ PCD may result from factors such as low gluten intake, sampling error, or suboptimal specimen orientation. However, the potential contribution of incomplete biopsy protocols to PCD misclassification has not been adequately explored, and even the most recent ESPGHAN Position Paper^31^ does not address the diagnostic impact of such limitations.

Although our retrospective design precludes causal inference, our findings strongly suggest that delayed adherence to biopsy guidelines at our tertiary center likely contributed to underdiagnosis and misclassification within the CD spectrum. Neglecting duodenal bulb sampling likely allowed subtle or localized mucosal lesions—hallmarks of USCD—to remain undetected, leading to potential misclassification as PCD. Consistent with these concerns, our data indicate that up to 77% of patients in our cohort could have been misclassified as PCD in the absence of systematic bulb sampling.

Given the suboptimal guideline adherence rates reported in the literature,^6^, ^30^ these findings may have broader implications for diagnostic accuracy worldwide.

Taken together, our findings underscore the critical importance of duodenal bulb biopsies for accurate histological assessment in pediatric CD, particularly for patients with TTG‐IgA <10× ULN, who remain ineligible for non‐biopsy diagnosis. While bulb sampling slightly increases procedural complexity, its omission carries far greater clinical risks: prolonged gluten exposure leading to persistent symptoms, growth impairment, micronutrient deficiencies, and increased risk of autoimmune comorbidities. Systematic bulb sampling should therefore be considered a cost‐effective measure to ensure early, accurate diagnosis and prevent long‐term complications.

Importantly, as emphasized in the Prague consensus report on CD transition, histologic reassessment is recommended at transition to adulthood when diagnosis was made without biopsy.^32^ This principle should equally apply to PCD cases diagnosed without targeted duodenal bulb biopsies.

Several limitations of this study should be acknowledged.

First, the retrospective design and variability in biopsy practices over the study period—including the gradual implementation of new diagnostic criteria—may have influenced diagnostic categorization.

Second, although follow‐up data supported the predictive model, clinical and serological outcomes were derived from routine practice rather than standardized prospective protocols, potentially introducing variability.

Third, the prediction of misclassification should be interpreted with caution, as true PCD cases may naturally progress to overt CD or show clinical benefit from a gluten‐free diet.^33^, ^34^

## 5 CONCLUSIONS

Our findings suggest a notable increase in the detection of USCD among pediatric patients undergoing EGD for CD diagnosis with low TTG‐IgA thresholds, exceeding the detection rates reported in previous literature.^13^, ^22^, ^23^ This reinforces the growing body of evidence advocating for a thorough and standardized histological approach, essential to ensure accurate diagnostic stratification across the CD spectrum, minimize the risk of delayed or missed diagnoses, and optimize clinical outcomes.

In an era where non‐biopsy diagnostic strategies are increasingly adopted, our data highlights that when histological evaluation is pursued, systematic sampling of both the distal duodenum and the duodenal bulb remains crucial.

Accordingly, we recommend that both pediatric and adult gastroenterologists critically re‐evaluate cases initially classified as PCD in the absence of bulb sampling and consider repeat endoscopy for proper re‐staging when clinically indicated, especially at transition to adult care.

## CONFLICT OF INTEREST STATEMENT

The authors declare no conflicts of interest.

## Supporting information


**Supplemental Figure S1** ‐ **Box plot of TTG‐IgA values in CD and USCD in DII+ Bulb group restricted to patients with TTG‐IgA < 10 × ULN**. The median, interquartile range, and outliers are shown. A statistically significant difference in TTG‐IgA levels was observed between CD and USCD. *Abbreviations: TTG‐IgA = tissue transglutaminase immunoglobulin A; CD = celiac disease; USCD = ultra‐short celiac disease; ULN = upper limit of normal; DII = distal duodenum*.

**Supplemental Table S1** – **Prevalence of clinical symptoms in patients with CD and USCD within the DII + Bulb group.** Only patients with available clinical history were included. *P* values refer to comparisons between CD and USCD groups for each symptom. A *p* value < 0.05 was considered statistically significant. *Abbreviations: CD = celiac disease; USCD = ultra‐short celiac disease; DII = distal duodenum*.

**Supporting file 1**.
