# Supplementary material for: High prevalence of ultra‐short celiac disease in children with low anti‐tissue transglutaminase immunoglobulin A levels: The risk of misdiagnosis without bulb biopsy
Source: J Pediatr Gastroenterol Nutr. 2026 May 25;83(2):296–303. doi: 10.1002/jpn3.70464 (PMC13446585; doi:10.1002/jpn3.70464)
Supplement: Supplementary file 1 [file JPN3-83-296-s001.docx]

|  |  | DIAGNOSIS | |  |
| --- | --- | --- | --- | --- |
|  | SYMPTOMS | CD | USCD | p-values |
| 1 | Abdominal pain | 40 (13.1%) | 46 (15.1%) | p = 0.18 |
| 2 | Asymptomatic | 35 (11.4%) | 18 (5.9%) | p = 0.03 |
| 3 | Poor growth | 22 (7.2%) | 31 (10.2%) | p = 0.07 |
| 4 | Familiarity | 21 (6.8%) | 18 (5.9%) | p = 0.87 |
| 5 | Diarrhea | 17 (5.8%) | 6 (1.9%) | p = 0.03 |
| 6 | Increased fatiguability | 6 (1.9%) | 9 (2.9%) | p = 0.31 |
| 7 | Mouth ulcers | 6 (1.9%) | 3 (0.9%) | p = 0.50 |
| 8 | Constipation | 3 (0.9%) | 7 (2.3%) | p = 0.02 |
| 9 | Anemia | 3 (0.9%) | 1 (0.3%) | p = 0.62 |
| 10 | Gassiness/flatulence | 3 (0.9%) | 6 (1.9%) | p = 0.31 |
| 11 | Dyspepsia | 1 (0.3%) |  | p = 1 |
| 12 | Dental enamel | 1 (0.3%) |  | p = 1 |
| 13 | Hematochezia | 1 (0.3%) |  | p = 1 |
| 14 | Epistaxis | 1 (0.3%) |  | p = 1 |
| 15 | Rash | 1 (0.3%) |  | p = 1 |
| 16 | Nausea/vomiting | 1 (0.3%) | 1 (0.3%) | p = 1 |
