# Supplementary material for: High prevalence of ultra‐short celiac disease in children with low anti‐tissue transglutaminase immunoglobulin A levels: The risk of misdiagnosis without bulb biopsy
Source: J Pediatr Gastroenterol Nutr. 2026 May 25;83(2):296–303. doi: 10.1002/jpn3.70464 (PMC13446585; doi:10.1002/jpn3.70464)
Supplement: Supplementary file 2 [file JPN3-83-296-s002.docx]

**Logistic Regression Models for Diagnostic Classification**

A binary logistic regression model was used to assess the association between increasing TTG-IgA and the likelihood of a definitive diagnosis of CD versus USCD. The dependent variable was diagnosis (1 = CD, 0 = USCD), and the independent variable was the TTG-IgA range, treated as an ordinal categorical variable (Range 1 [TTG-IgA < 2 × ULN], Range 2 [TTG-IgA 2-5 × ULN], Range 3[TTG-IgA 5-10 × ULN]). The odds ratio (OR) with 95% confidence intervals (CIs) and p- values were calculated. A p-value < 0.05 was considered statistically significant.

To assess the likelihood of missed USCD diagnoses in patients with PCD within the DII group, we applied a logistic regression model, using TTG-IgA as the predictor variable. The model was trained using data from patients who had both DII + Bulb biopsies and was then used to predict the likelihood of USCD in patients with histology limited to DII. The model output provided a probability score ranging from 0 to 1. To establish an optimal threshold for classification, the cutoff was chosen based on the highest Youden Index, which maximizes the combined sensitivity and specificity. A threshold of 0.52 was identified, meaning that patients with a predicted probability ≥ 0.52 were classified as USCD, while those with a score below this threshold were classified as CD.
